# Xuebijing injection in the treatment of COVID-19: An update on clinical studies, potentially active metabolites and mechanisms

**DOI:** 10.3389/fphar.2025.1667022

**Published:** 2026-01-14

**Authors:** Ziyan Zhang, Xinyu Li, Jiping Zhou, Yupeng Li

**Affiliations:** 1 Department of Emergency, The Second Affiliated Hospital of Liaoning University of Traditional Chinese Medicine, Shenyang, Liaoning, China; 2 Hong Kong Baptist University, Hong Kong, Hong Kong SAR, China; 3 Department of Internal, Kelan County Traditional Chinese Medicine Hospital, Xinzhou, Shanxi, China

**Keywords:** angiotensin converting enzyme 2, COVID-19, severe pneumonia, traditional Chinese medicine, Xuebijing injection

## Abstract

**Introduction:**

Coronavirus disease 2019 (COVID-19) is an epidemic respiratory disease caused due to the infection of severe acute respiratory syndrome coronavirus 2 (SARS-CoV-2). In China, the National Health Commission of China announced that patients with COVID-19 who were treated with traditional Chinese medicines (TCMs) combined with antiviral drugs effectively alleviated their symptoms and recovered. Among these TCMs, Xuebijing (XBJ) injection plays an important role in the treatment of patients with COVID-19. However, this was a puzzle that what will be the clinical efficacy and safety of XBJ injection for COVID-19 treatment, and what are the potential mechanisms behind XBJ injection?

**Methods:**

To search for articles on “Xuebijing injection in the treatment of COVID-19” in PubMed, use the following query: (Xuebijing injection OR Xuebijing) AND (COVID-19 OR SARS-CoV-2 OR severe pneumonia). We added filters for “Clinical Trial,” “Randomized Controlled Trial,” or “Review” to focus on specific study types, and limit the search to recent years (2010–2025) and English-language articles for more targeted results.

**Results:**

XBJ injection in combination with regular therapy has been shown to improve overall efficacy, reduce 28-day mortality, improve lung CT recovery and reduce pro-inflammatory markers in patients with COVID-19. The high affinity for angiotensin converting enzyme 2, inhibition of neutrophil extracellular trap release and prevention of cell death and inflammation may be the main molecular mechanisms of XBJ injection in the treatment of COVID-19.

**Conclusion:**

This review synthesizes the current evidence on the clinical efficacy and safety of XBJ injection in the treatment of COVID-19. Our analysis indicates that XBJ injection, when used in combination with standard therapy, significantly improves overall efficacy, reduces 28-day mortality, enhances lung CT recovery, and decreases pro-inflammatory markers such as C-reactive protein (CRP) and interleukin-6 (IL-6). These findings suggest that Xuebijing injection is a promising adjunctive treatment for COVID-19, particularly in severe cases, although it must be confirmed through rigorous pharmacological and clinical studies.

## 1 Introduction

Coronavirus disease 2019 (COVID-19) is an epidemic respiratory disease caused by severe acute respiratory syndrome coronavirus 2 (SARS-CoV-2) infection (Yüce et al., 2021; Lino et al., 2025). COVID-19 is highly contagious, characterized by atypical clinical symptoms and easily missed diagnosis, which seriously affects the socio-economic development and health of the population (Hadj, 2022; de Melo et al., 2025a). As of October 2021, the number of confirmed COVID-19 infections has exceeded 242 million globally, with nearly five million deaths reported across multiple countries, according to the World Health Organization (WHO) and other international health agencies (Sharma et al., 2021). Although COVID-19 vaccination can partially reduce the incidence and severity of the disease, it cannot prevent infection (Fernandes et al., 2022). In the early stage of COVID-19, there are mainly fever, fatigue, dry cough and other manifestations, and such as upper respiratory tract symptoms (nasal congestion, runny nose, etc.) is less common; in addition, some patients will also have varying degrees of hypoxia. Many patients will develop respiratory distress 1 week after the onset of the disease and may even progress to septic shock, acute respiratory distress syndrome (ARDS), or even death (Szafran et al., 2023). This pandemic has highlighted the significant role of infectious agents, including viruses and bacteria, in the progression and severity of various diseases. Historically, we have faced numerous disease outbreaks, such as the West Nile Virus, sexually transmitted infections, measles, malaria, and others, each posing unique challenges to public health (Cilloniz et al., 2016; Sharma et al., 2022; Singh et al., 2022; Heidecke et al., 2025). Therefore, it is necessary to provide active treatment and achieve early detection, early isolation, and early treatment (Radcliffe et al., 2023).

SARS-CoV-2 is a coronavirus of the genus β, with an envelope, and the particles are presented as elliptical or round, in addition, most of them are polymorphic, with a general diameter of 60–140 nm; and in terms of genetic characterization, it is different from SARSr-CoV (Chen et al., 2023; Shahi et al., 2023). SARS-CoV-2 has been reported to be >85% homologous to the bat SARS-like coronavirus (bat-SL-CoVZC45) (Cilibrasi and Vitányi, 2022; de Melo et al., 2025b). Some studies suggest that SARS-CoV-2 is sensitive to heat and ultraviolet light and can be killed by lipid solvents such as ethanol, 75% ethanol, and peroxyacetic acid (Mathew et al., 2023). Drug repurposing has emerged as a vital strategy in addressing emerging and challenging diseases, including COVID-19. By identifying new therapeutic uses for existing drugs, this approach can accelerate the development of treatments and reduce the time and cost associated with drug discovery (Singh et al., 2022). Additionally, computational approaches have been employed to predict the role of pathogens in various diseases, such as the targeting of *Mycoplasma* hominis proteins in prostate cancer etiology (Heidecke et al., 2025; Khan et al., 2017; Khan et al., 2016a) and the implications of *Helicobacter pylori* proteins in gallbladder cancer (Khan et al., 2016b). Antiviral drugs are mostly used to treat COVID-19, such as ritonavir and α-interferon nebulization, but the therapeutic effect is not exact. In China, the National Health Commission of China announced that patients with COVID-19 who were treated with traditional Chinese medicine (TCM) combined with antiviral drugs had their symptoms effectively alleviated and recovered (Liu, 2020; Zhang et al., 2025). Among these TCMs, Xuebijing (XBJ) injection plays a critical role in the treatment of patients with COVID-19, which is recommended by the National Health Commission of China to treat severe and critical cases of COVID-19 (China NHCotPsRo, 2022).

XBJ injection is a Chinese botanical medicine developed by Prof. Jinda Wang based on the principle of “Three Evidences and Three Methods” and the theory of “simultaneous treatment of bacteria and virus” and Xuefu Zhuyu decoction with the effects of dispersing toxins, resolving blood stasis, and activating collaterals (Zhang et al., 2023; Liao et al., 2025). XBJ injection is composed of five traditional Chinese botanical drugs, including *Angelica sinensis* (Oliv.) Diels (*Angelicae sinensis* Radix, Danggui), *Ligusticum chuanxiong* Hort. (Chuanxiong rhizoma, Chuanxiong), *Paeonia lactiflora* Pall. (*Paeoniae radix* Rubra, Chishao), *Carthamus tinctorius* L. (Carthami Flos, Honghua), and *Salvia miltiorrhiza* Bge. (Salviae miltiorrhizae Radix Et Rhizoma, Danshen) (Li et al., 2021; Cui et al., 2025). These herbs are known for their anti-inflammatory, antioxidant, and antiviral properties. On 12 April 2020, the National Drug Administration (NMPA) of China approved the use of XBJ for “COVID-19 pneumonia with severe, critically ill systemic inflammatory response syndrome or/and multi-organ failure”. However, what is the clinical efficacy and safety of XBJ injection for COVID-19 treatment and what are the potential effects behind XBJ injection? This review summarizes the basic and clinical research progress of XBJ injection for COVID-19 treatment to date, and expects to provide clinical evidence for the effects and safety of clinical use of XBJ injection for COVID-19 patients.

## 2 Potential related metabolites of XBJ injection

To search for articles on “Xuebijing injection in the treatment of COVID-19,” we conducted a comprehensive literature search using PubMed. The query used was: (Xuebijing injection OR Xuebijing) AND (COVID-19 OR SARS-CoV-2 OR severe pneumonia). We added filters for “Clinical Trial,” “Randomized Controlled Trial,” or “Review” to focus on specific study types and limited the search to recent years (2010–2025) and English-language articles for more targeted results. While we acknowledge that other databases such as Embase, Web of Science, Scopus, Cochrane Library, and CNKI may also contain relevant studies, we chose to focus on PubMed due to its extensive coverage of high-quality biomedical literature, ease of access, and the efficiency of its search tools. This approach allowed us to efficiently identify and analyze a substantial number of relevant studies for our review. In the pharmacological studies reviewed, the dose range tested varied from 10 μM to 100 µM for *in vitro* studies and 10 mg/kg to 100 mg/kg for *in vivo* studies. The minimal active concentration was reported to be 10 µM in several *in vitro* studies. The models used included HEK-293T cells, Vero-E6 cells, and various animal models such as mice and rats. Both positive and negative controls were used in these studies, with durations ranging from 24 h to 14 days. The type of extract used was specified in each study, and basic pharmacological data were provided to assess the claims.

Artificial intelligence (AI) and bioinformatics play a pivotal role in managing newly arising diseases and epidemics. These technologies enable the rapid identification of potential drug targets and the prediction of pathogen behavior, facilitating the development of effective treatment strategies (Zhou et al., 2020). More than 100 metabolites have been discovered in XBJ injection as it is a chemically complex botanical injection (Chen et al., 2025). Among them, danshensu, hydroxysafflor yellow A, paeoniflorin, ferulic acid, and senkyunolide I have been characterized as the major related metabolites of XBJ injection (Li et al., 2019; Li et al., 2018; Chen et al., 2010; Zuo et al., 2019; Chen et al., 2012). Advanced analytical techniques such as ultra-high performance liquid chromatography-high resolution hybrid quadrupole-orbitrap mass spectrometry (UHPLC-Q-Orbitrap MS) and ultra-high performance liquid chromatography-quadrupole time-of-flight mass spectrometry (UPLC-Q-TOF-MS) have further characterized additional metabolites, such as gallic acid, 5-hydroxymethylfurfural, matrine, tanshinone IIA, protocatechuic aldehyde, caffeic acid, galuteolin, apigenin, benzoylpaeoniflorin, kaempferol, and ethyl ferulate (Sun et al., 2017; Zuo et al., 2017a; Huang et al., 2013; Ouyang and He, 2018; Zuo et al., 2017b; Ji et al., 2010; Huang et al., 2011). These metabolites have demonstrated potential pharmacological activities *in vitro* or in animal models, including inhibition of inflammation, prevention of cell death, and enhancement of immune function, though their clinical relevance remains to be established.

Through pharmacokinetic investigation, 10 phthalides and 17 danshen catechols, including nine major metabolites, have been discovered in sepsis treatments (Zhang et al., 2018; Li et al., 2016). Meanwhile, 18 monoterpene glycosides have been discovered by LC/TOF-MS in XBJ antiseptic injection in humans and rats (Cheng et al., 2016). In addition, one of the most pharmaceutically relevant metabolites in XBJ injection for the treatment of traumatic brain injury, hydroxysafflor yellow A, has been shown to cross the blood-brain barrier, suggesting the potential use of XBJ injection in brain diseases (Sheng et al., 2020). A summary of the potential related metabolites of XBJ injection is shown in Table 1. In addition, Li et al. recently reported an aggregation-induced emission sensor combined with UHPLC-Q-TOF/MS that can rapidly identify anticoagulants from XBJ injection (Li et al., 2023). The total inhibition rate of the six mixed standards was approximately 60% of the inhibition rate of XBJ injection, providing a novel, inexpensive, and simple method for monitoring thrombin activity and screening agents from XBJ injection. The following is a list of important active metabolites and their underlying mechanisms (Table 2). While analytical methods such as UPLC-Q-TOF-MS and RP-HPLC are essential for identifying and quantifying metabolites, they do not provide direct evidence of therapeutic activity. Therefore, we have focused on studies that provide *in vitro*, *in vivo*, and clinical evidence for the activity of these metabolites.

**TABLE 1:** Related metabolites of XBJ injection.

Disease	Methods	Metabolites	Potential related metabolites	References
Sepsis	Literature-mining and *in vitro* experiments	12	Hydroxysafflor yellow A, paeoniflorin, oxypaeoniflorin, albiflorin, senkyunolides I and G, tanshinol, salvianolic acid B, protocatechuic acid, ferulic acid, senkyunolide I-7-O-β-glucuronide, and 3-O-methyltanshinol	Li et al. (2019)
N/A	UHPLC-Q exactive hybrid quadrupole-orbitrap high-resolution MS	162 (including 38 major metabolites)	Gallic acid, 5-hydroxymethylfurfural, matrine, salvianic acid A sodium, tanshinol, protocatechuic acid, protocatechuic aldehyde, tetramethylpyrazine, catechin, chlorogenic acid, caffeic acid, oxypaeoniflorin, hydroxysafflor yellow A, albiflorin, paeoniflorin, ferulic acid, rutin, hyperin, quercetin, luteolin-O-glc, senkyunolide I/H, rosmarinic acid, crocin I, salvianolic acid B, luteolin, salvianolic acid A, naringenin, paeonol, apigenin, benzoylpaeoniflorin, kaempferol, ethyl ferulate, ligustilide, butylidenephthalide, tanshinone I, cryptotanshinone, levistolide A, and tanshinoneαA	Sun et al. (2017)
N/A	HPLC-DAD	5	Danshensu, hydroxysafflor yellow A, paeoniflorin, ferulic acid, and senkyunolide I	Li et al. (2018)
N/A	UHPLC-Q-Orbitrap MS	30	Gallic acid, 5-hydroxymethylfurfural, sodium danshensu, tanshinol, protocatechuic acid, hydroxysafflor yellow A, oxypaeoniflorin, chlorogenic acid, catechinic acid, hyperoside, rutin, albiflorin, protocatechuic aldehyde, caffeic acid, galuteolin, salvianolic acid B, rosmarinic acid, ferulic acid, salvianolic acid a, senkyunolide I, quercetin, luteolin, benzoylpaeoniflorin, apigenin, naringenin, ethyl ferulate, paeonol, butylidenephthalide, cryptotanshinone, and tanshinone IIA	Zuo et al. (2017a)
N/A	UPLC-Q-TOF-MS	13	Uridine, gallic acid, guanosine, danshensu, protocatechualdehyde, oxypaeoniflorin, hydroxysafflor yellow A, paeoniflorin, ferulic acid, safflor yellow A, senkyunolide I, senkyunolide H, and salvianolic acid B	Huang et al. (2013)
N/A	RP-HPLC	5	hydroxysafflor yellow A, paeoniflorin, ferulic acid, benzoic acid, and danshensu	Chen et al. (2010)
Sepsis	Pharmacokinetic investigation	10 Phthalides	Senkyunolide I, senkyunolide H, senkyunolide G, senkyunolide N, 3-hydroxy-3-n-butylphthalide, Z-6,7-epoxyligustilide, 6,7-dihydroxyligustilide, senkyunolide A, senkyunolide J, and 4-hydroxy-3-nbutylphthalide	Zhang et al. (2018)
Sepsis	Pharmacokinetic investigation	17 Danshen catechols (including 9 major metabolites)	Tanshinol, salvianolic acid B, protocatechuic acid, isosalvianolic acid C, rosmarinic acid, protocatechuic aldehyde, salvianolic acid D, salvianolic acid C, and lithospermic acid	Li et al. (2016)
Sepsis	LC/TOF-MS	18 monoterpene glycosides	Mudanpioside F, 1-O-β-D-glucopyranosyl-paeonisuffrone, desbenzoylpaeoniflorin, albiflorin, paeoniflorin, oxypaeoniflorin, oxypaeoniflorin isomer, ortho-oxypaeoniflorin, mudanpioside E, 6′-O-galloyl-desbenzoylpaeoniflorin, benzoylpaeoniflorin, benzoyloxypaeoniflorin, mudanpioside C, mudanpioside J, galloylpaeoniflorin, isomer of galloylpaeoniflorin or galloylalbiflorin, and galloyloxypaeoniflorin	Cheng et al. (2016)
N/A	UHPLC-Q-Orbitrap HRMS	4	Hydroxysafflor yellow A, oxypaeoniflorin, ferulic acid, and benzoylpaeoniflorin	Zuo et al. (2019)
N/A	HPLC-MS/MS	9	Ferulic acid, benzoylpaeoniflorin, danshensu, chlorogenic acid, rosmarinic acid, hydroxy-methyl safflower yellow A, paeoniflorin, albiflorin, and oxypaeoniflorin	Ouyang and He (2018)
N/A	UHPLC-Q-Orbitrap HRMS	12	Gallic acid, hydroxysafflor yellow A, oxypaeoniflorin, chlorogenic acid, rutin, luteoloside, albiflorin, hyperoside, rosmarinic acid, ferulic acid, salvianolic acid A, and benzoylpaeonif-lorin	Zuo et al. (2017b)
N/A	HPLC-MS/MS	4	Danshensu, hydroxysafflor yellow A, paeoniflorin, and ferulic acid	Chen et al. (2012)
Traumatic brain injury	LC-MS/MS	1	Hydroxysafflor yellow A	Sheng et al. (2020)
N/A	UPLC-Q-TOF-MS	11	Paeoniflorin, senkyunolide I, safflor yellow A, danshensu, uridine, ferulic acid, salvianolic acid B, uridine, senkyunolide H, gallic acid, and protocatechuic aldehyde	Ji et al. (2010)
N/A	HPLC-ESI-MS	21	Uridine, gallic acid, guanosine, danshensu, protocatechuic aldehyde, hydroxysafflor yellow A, oxypaeoniflorin, 6-hydroxykaemferol 3,6,7-tri-O-β-D-glucopyranoside, 6-hydroxykaemferol 3,6-Di-O-β-D-glucopyranosyl-7-O-β-D-glucuronopyranoside, caffeic acid, albiflorin, 4′,5,6,7-Tetrahydroxyflavanone 6,7-Di-O-β-D-glucopyranoside, paeoniflorin, ferulic acid, galloylpaeoniflorin, anhydrosafflor yellow B, safflor yellow A, senkyunolide I, senkyunolide H, salvianolic acid B, and benzoylpaeoniflorin	Huang et al. (2011)
N/A	UPLC-QTOF-MS	5	ML 334, Deoxynyboquinone, Tanshinone IIA, Luteolin, Baicalein	Lin et al. (2025)

UPLC-Q-TOF-MS, ultra-high-performance liquid chromatography-quadrupole time-of-flight mass spectrometry; UHPLC-Q-Orbitrap HRMS, ultra-high performance liquid chromatography-Q, executive hybrid quadrupole-orbitrap high-resolution accurate mass spectrometry; UHPLC-Q-Orbitrap MS, ultra-high performance liquid chromatography-Q, executive hybrid quadrupole-orbitrap mass spectrometry; HPLC-ESI-MS, high-performance liquid chromatography electrospray ionization-tandem mass spectrometry.

**TABLE 2:** A list of important active metabolites and their underlying mechanisms.

Metabolite names	Chemical structures	Molecular mechanisms
Danshensu	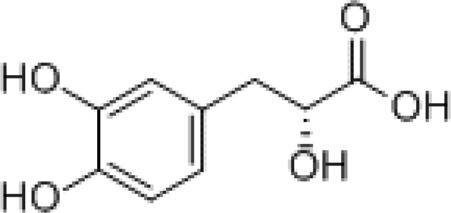	Preventing SARS-CoV-2 from entering certain cells
Hydroxysafflor yellow A	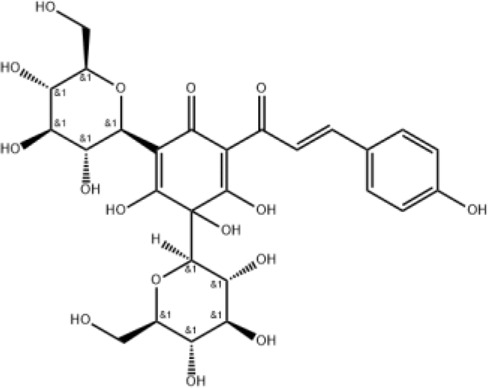	Inhibiting TLR4-dependent pathways and activating antioxidant mechanisms
Paeoniflorin	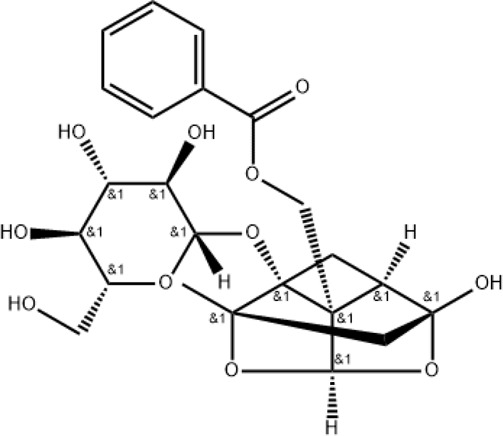	Regulating MAPK/NF-κB, PI3K/AKT/mTOR, and JAK2/STAT3 pathways
Ferulic acid	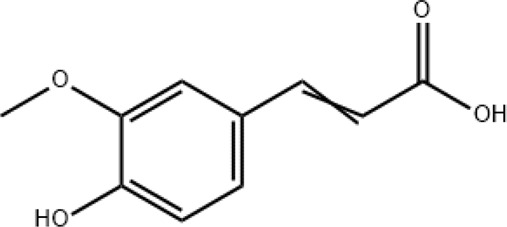	Targeting autophagy and protecting against intracerebral hemorrhages
Senkyunolide I	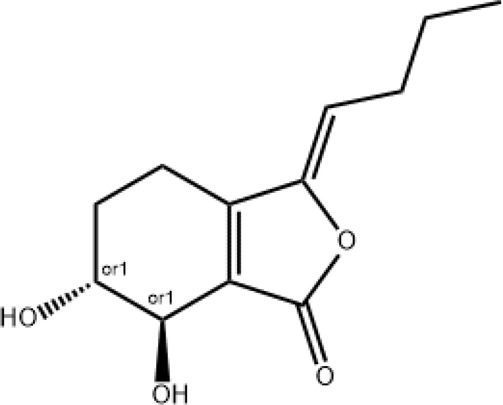	Preventing the formation of neutrophil extracellular traps
Baicalein	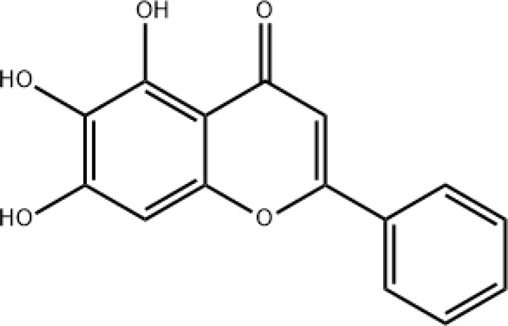	Targeting the KEAP1 protein, thereby activating the NRF2/KEAP1 signaling pathway

### 2.1 Danshensu

Danshensu is a pure molecule derived from the root of *S. miltiorrhiza* and is also one of the main active metabolites in XBJ injection (Zhang et al., 2019). *In vitro* studies have shown that danshensu inhibits the entry of SARS-CoV-2 into ACE2-overexpressed cells (Bourgonje et al., 2020). Both oral and intravenous pretreatment with danshensu dose-dependently ameliorated the pathological changes in mice infected with pseudotyped SARS-CoV-2 (Wang et al., 2022). Meanwhile, a study by Wang et al. showed that danshensu is a covalent inhibitor of 3-chymotrypsin-like protease against SARS-CoV-2 (Wang et al., 2022).

### 2.2 Hydroxysafflor yellow A

Hydroxysafflor yellow A can be extracted from the flower of *C. tinctorius L* and is also one of the main active metabolites in XBJ injection (He et al., 2021). Hydroxysafflor yellow A could reduce pathological changes, pulmonary edema, pulmonary vascular permeability, levels of inflammatory mediators, and myeloperoxidase (MPO) activity in mice with acute lung injury (ALI) induced by lipopolysaccharide (LPS) or bleomycin (Wu et al., 2012; Sun et al., 2010). The mechanisms involve inhibition of TLR4-dependent MAPK and NF-κB pathways (Liu et al., 2014). In addition, hydroxysafflor yellow A (15 mg/kg) can inhibit lung injury in an oleic acid-induced ALI rat model by activating antioxidant enzymes and inactivating the inflammatory response via the cAMP/PKA pathway (Wang et al., 2013).

### 2.3 Paeoniflorin

Paeoniflorin can be extracted from *Paeoniae Radix Rubra* (Chishao in Chinese) and is also one of the main active metabolites in XBJ injection. It has various pharmacological effects and has been shown to protect mice against LPS-induced ALI (Zhou et al., 2011). The anti-inflammatory mechanism of paeoniflorin is associated with the regulation of MAPK/NF-κB, PI3K/AKT/mTOR, and JAK2/STAT3 pathways (Zhang and Wei, 2020). However, the mechanisms of paeoniflorin in the treatment of COVID-19 remain to be elucidated.

### 2.4 Ferulic acid

Ferulic acid (4-hydroxy-3-methoxycinnamic acid) is a phenolic bioactive metabolite belonging to a group of hydroxycinnamates and is also one of the main active metabolites in XBJ injection. It has high antioxidant activity, antibacterial, anti-inflammatory, neuro- and photoprotective, antidiabetic, anticancer, and skin-whitening effects (Stompor-Gorący and Machaczka, 2021). Salman et al. investigated the effects of ferulic acid on anti-SARS-CoV-2 (Salman et al., 2020). A preclinical finding showed that ferulic acid attenuated COVID-19 by targeting autophagy (Pang et al., 2022). In addition, ferulic acid has been reported to have neuroprotective potentials against COVID-19 intracerebral hemorrhage (Dong et al., 2023).

### 2.5 Senkyunolide I

Senkyunolide I can be extracted from *Ligusticum* Chuanxiong hort and is also one of the main active metabolites in XBJ injection (Li et al., 2012). Senkyunolide I has the effects of anti-migraine, anti-inflammation, anti-oxidation and sedation (Hu et al., 2016; Hu et al., 2015; Wang et al., 2011). In a study be Zha et al., Senkyunolide I protected against ALI via inhibiting formation of neutrophil extracellular traps in a murine model of cecal ligation and puncture (Zha et al., 2021). However, the mechanisms of paeoniflorin in the treatment of COVID-19 remain to be elucidated.

### 2.6 Baicalein

Baicalein can be extracted from Honghua/Chishao. According to Lin et al. (Lin et al., 2025), baicalein plays an important role in treating SARS-CoV-2 infection. Baicalein targets the KEAP1 protein, thereby activating the NRF2/KEAP1 signaling pathway. This pathway increases the expression of antioxidant enzymes, such as heme oxygenase-1. This enhances the cells’ antioxidant capacity and reduces oxidative stress damage.

However, several metabolites identified in XBJ injection, such as ferulic acid, are known to be pan-assay interfering substances (PAINS) in *in vitro* assays (Sifontes-Rodríguez et al., 2025). PAINS are compounds that exhibit activity across a wide range of assays, often due to nonspecific interactions rather than specific biological activity. This can lead to false-positive results in high-throughput screening and other *in vitro* studies, potentially inflating the perceived effects of certain metabolites. The presence of PAINS in XBJ injection raises important questions about the validity of conclusions drawn from *in vitro* studies. While these compounds may show promising results in laboratory settings, their true therapeutic potential must be validated through *in vivo* and clinical studies to avoid false positives. For instance, ferulic acid has been shown to exhibit antioxidant and anti-inflammatory properties in *in vitro* assays. However, its effectiveness in a biological context may be limited by its nonspecific interactions, which could interfere with accurate assessment of its therapeutic potential. To critically assess the impact of PAINS on the interpretation of the effects of XBJ injection, the therapeutic potential of metabolites identified *in vitro* should be confirmed through *in vivo* animal models and clinical trials. This step is crucial to differentiate between true biological activity and nonspecific interactions caused by PAINS. Moreover, researchers should be cautious about selectively reporting positive results from *in vitro* studies without considering the potential for PAINS interference. A comprehensive evaluation of both positive and negative findings is necessary to provide a balanced view of the metabolite’s effects. To mitigate the impact of PAINS, the use of orthogonal assays that are less susceptible to nonspecific interactions is recommended. This approach can help confirm the specificity of the observed effects and provide more reliable data. When reviewing the literature on XBJ injection, it is important to critically evaluate studies that rely solely on *in vitro* findings. The presence of PAINS should be considered as a potential confounding factor, and conclusions should be drawn with caution until *in vivo* and clinical data are available.

In summary, although *in vitro* studies have identified several metabolites in XBJ injection with potential therapeutic effects, the presence of PAINS necessitates a cautious approach. Further confirmation through *in vivo* and clinical investigations is essential to validate the true effects of these metabolites and to ensure that the conclusions drawn are not influenced by nonspecific interactions.

## 3 Research on material basis

XBJ injection is composed of five traditional Chinese medicinal materials. The main components of these raw materials include danshensu, hydroxysafflor yellow A, paeoniflorin, ferulic acid, and senkyunolide I, etc. These components have higher content in the medicinal materials and possess clear pharmacological activities. During the preparation process of XBJ injection, the transfer and degradation of the main components are analyzed. Through high-performance liquid chromatography (HPLC) and mass spectrometry (MS), some components would undergo a certain degree of degradation during the extraction and purification processes. For example, the water-soluble components in Salvia miltiorrhiza may partially transform into other compounds during the high-temperature extraction process (Liu et al., 2025). By optimizing the extraction process, the degradation of components is minimized and a component traceability chain is established to ensure that the changes of components from raw materials to finished products can be effectively tracked. A component traceability chain from “raw materials-intermediates-finished products” is established. By analyzing the components at each stage, the patterns of component changes during the preparation process are clarified. The establishment of this traceability chain helps us to better control the product quality and ensure the stability and consistency of the components in XBJ injection.

## 4 Potential targets of XBJ injection

**FIGURE 1:**
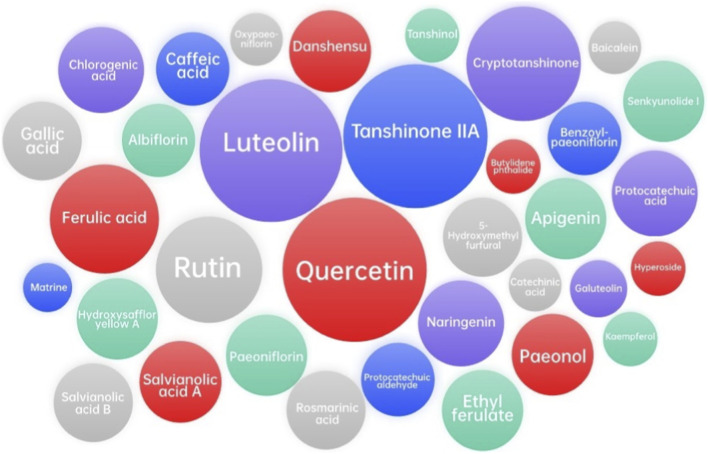
Major related metabolites from network analysis and molecular docking analysis between XBJ injection and COVID-19. However, further confirmation through *in vivo* and clinical investigations is essential to validate the true effects of these metabolites and to ensure that the conclusions drawn are not influenced by nonspecific interactions.

Network analysis and molecular docking analysis approaches were used to investigate the active metabolites, potential molecular targets, and predictive mechanisms of XBJ injection (Lin et al., 2025; Tianyu and Liying, 2021; Xing et al., 2020; Niu et al., 2021; Zheng et al., 2020; Tao and Jiming, 2022). These studies identified the potential related metabolites using online databases, including Traditional Chinese Medicine Systems Pharmacology Database and Analysis Platform (TCMSP, http://tcmspw.com/tcmsp.php), Encyclopedia of Traditional Chinese Medicine (ETCM, http://www.nrc.ac.cn:9090/ETCM/index.php/Home/Index/index.html), PubChem (https://pubchem.ncbi.nlm.nih.gov/), etc., and found that luteolin and quercetin might be the main metabolites in XBJ injection, as well as cryptotanshinone, ferulic acid, rutin, and tanshinone IIA, although they are still pending experimental validation. The potential targets of XBJ injection could be tumor necrosis factor (TNF), mitogen-activated protein kinase 1 (MAPK1), Caspase-3 (CASP3), epidermal growth factor receptor (EGFR), interleukin-1β (IL1B), c-Jun (JUN), mitogen-activated protein kinase 8 (MAPK8), myeloperoxidase (MPO), prostaglandin-Endoperoxide Synthase 2 (PTGS2), nuclear factor kappa-b subunit RelA (RELA), and tumor protein p53 (TP53). Zhao et al. suggested that the potential molecular mechanisms by which XBJ injection inhibits COVID-19 are by acting on AKT serine/threonine kinase 1 (AKT1) (Tianyu and Liying, 2021). Xing et al. found that TNF, MAPK1, and interleukin-6 (IL-6) may be the key to the treatment of COVID-19 (Xing et al., 2020). Niu et al. suggested that XBJ could inhibit COVID-19 by down-regulating IL-6 (Niu et al., 2021). Zheng et al. showed that glyceraldehyde-3-phosphate dehydrogenase (GAPDH), albumin (ALB), TNF, EGFR, and MAPK1 might be involved in the regulation of COVID-19 by XBJ injection (Zheng et al., 2020). For the treatment of COVID-19-induced acute respiratory distress syndrome (ARDS), 56 targets of XBJ injection have been identified, among which AKT1, TNF, CASP3, and signal transducer and activator of transcription 3 (STAT3) are the main targets (Tao and Jiming, 2022). The following gene ontology (GO) biological process enrichment analysis and Kyoto Gene and Genome Encyclopedia (KEGG) enrichment analysis clarified some important pathways. Interestingly, the TNF signaling pathway seemed to play a critical role in XBJ injection treatment for COVID-19, which deserves further investigation. The results of network analysis and molecular docking analysis of XBJ injection in the above five articles are shown in Table 3 and Figure 1.

**TABLE 3:** Network analysis and molecular docking analysis between XBJ injection and COVID-19.

Classification*	Predicted metabolites	Predicted target molecules**	GO biological process enrichment analysis***	KEGG enrichment analysis***
Existing in 5 articles	Luteolin Quercetin Tanshinone IIA	TNF	-	-
Existing in 4 articles	Cryptotanshinone Ferulic acid Rutin	MAPK1	response to lipopolysaccharide	TNF signaling pathway
Existing in 3 articles	5-Hydroxymethyl furfural Albiflorin Apigenin Benzoylpaeoniflorin Caffeic acid Chlorogenic acid Danshensu Ethyl ferulate Gallic acid Hydroxysafflor yellow A Naringenin Paeoniflorin Paeonol Protocatechuic acid Protocatechuic aldehyde Rosmarinic acid Salvianolic acid A Salvianolic acid B Senkyunolide I	CASP3 EGFR IL1B JUN MAPK8 MPO PTGS2 RELA TP53	regulation of inflammatory response regulation of protein serine/threonine kinase activity response to molecule of bacterial origin	Hepatitis C
Existing in 2 articles	Butylidenephthalide Catechinic acid Galuteolin Hyperoside Kaempferol Matrine Oxypaeoniflorin Tanshinol, Baicalein	AKT1 AR BCL2L1 CA2 CASP8 CAT CCL2 CTNNB1 EGF ESR1 FGF2 GSK3B IFNG IL2 IL6 MAPK14 MAPK3 MMP1 PPARG SELE STAT3 KEAP1	cell chemotaxis cellular response to biotic stimulus cytokine activity regulation of leukocyte migration regulation of neuron death response to oxidative stress response to reactive oxygen species	Apoptosis C-type lectin receptor signaling pathway HIF-1 signaling pathway IL-17 signaling pathway

*The five articles include references (Tianyu and Liying, 2021; Xing et al., 2020; Niu et al., 2021; Zheng et al., 2020; Tao and Jiming, 2022). **The molecules in bold represent the focused targets in the five articles. ***Refence (Niu et al., 2021) is excluded due to the unavailable data. GO, gene ontology; KEGG, kyoto gene and genome encyclopedia.

The network analysis reveals that XBJ injection might targets multiple key molecular pathways. By modulating the activity of proteins such as TNF, MAPK1, CASP3, EGFR, IL1B, MPO, and RELA, XBJ injection can potentially reduce inflammation, prevent excessive immune activation, and promote tissue repair. These network-based predictions offer hypothetical insights into possible modes of action, which require further experimental and pharmacological validation.

### 4.1 Finding of basic studies

Pharmacological studies have shown that XBJ injection can activate the phagocytosis function of the reticuloendothelial system and also strengthen the humoral immune function; thus, it can treat bacteria and viruses, activate blood circulation, relieve blood congestion, and clear fever and toxins (Cheng and Yu, 2021). In addition, XBJ injection can inhibit the elevation of serum tumor necrosis factor alpha (TNF-α) levels caused by endotoxin and promote the decrease of C-reactive protein (CRP) levels, which is a sensitive indicator commonly used to evaluate the degree of inflammatory response and shows a significant positive correlation with leukocyte levels (Song et al., 2020). When the individual is attacked by SARS-CoV-2, CRP levels tend to rise, leading to severe inflammation and exacerbation of COVID-19.

**FIGURE 2:**
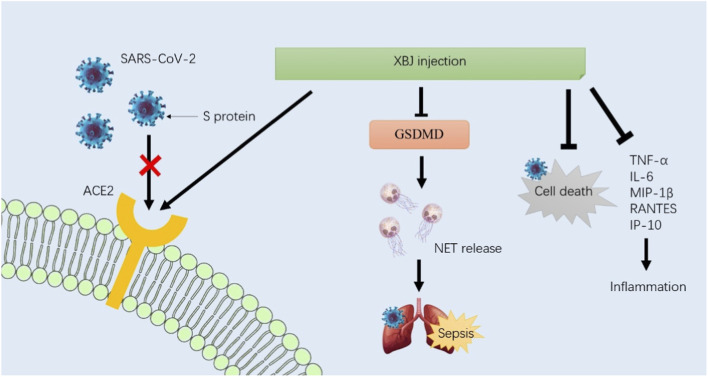
Based on the basic studies, the high affinity for ACE2, inhibition of NET release, and prevention of cell death and inflammation may be the main molecular mechanisms of XBJ injection in the treatment of COVID-19. Future research should further elucidate these mechanisms and identify patient subgroups that are most likely to benefit from XBJ injection.

The spike protein (S protein) is responsible for coronavirus entry into host cells. Because angiotensin converting enzyme 2 (ACE2) can bind to the receptor binding domain (RBD) of the S protein, it was identified as the critical functional receptor for SARS-CoV-2, making it the important intervention target for COVID-19. The metabolites of anhydrosafflor yellow B, salvianolic acid B, and rutin in XBJ injection showed high affinity for ACE2 in the treatment of COVID-19, suggesting that it may be a molecular mechanism of XBJ injection for COVID-19 treatment (Zhang and Qi, 2023). The release of neutrophil extracellular traps (NETs) is a major cause of organ failure and mortality in sepsis, as well as in lung injury during COVID-19. In septic mouse models, XBJ injection reduced neutrophil recruitment and chemokines, including CSF-3, CSF-2, CXCL-3, and CXCL-2, in the lungs. It could also inhibit NET formation by reducing the expressions of citrullinated histone H3 (CitH3), MPO, and neutrophil elastase (NE) and normalizing sepsis-induced overexpression of targeting gasdermin D (GSDMD) (Shang et al., 2022). In addition, XBJ injection could significantly protect cells from SARS-CoV-2-induced cell death and inhibit the average size and number of plaques *in vitro* (Ma et al., 2020). It inhibited the release of inflammatory mediators, including TNF-α, IL-6, MIP-1β, RANTES, and IP-10, induced by SARS-COV-2 in Huh-7 cells. Taken together, the high affinity for ACE2, inhibition of NET release, and prevention of cell death and inflammation may be the main molecular mechanisms of XBJ injection in the treatment of COVID-19 (Figure 2).

In summary, XBJ injection exerts its therapeutic effects through a multi-target mechanism that includes inhibition of viral entry, reduction of inflammation, protection against cell death, and promotion of tissue repair. These mechanisms collectively contribute to the management and treatment of COVID-19, particularly in severe cases, although it must be confirmed through rigorous pharmacological and clinical studies. Future research should further elucidate these mechanisms and identify patient subgroups that are most likely to benefit from XBJ injection.

## 5 Findings of clinical studies

Several randomized controlled trials (RCTs) have been conducted to evaluate the therapeutic effects of XBJ injection in the treatment of COVID-19. In patients with confirmed and suspected COVID-19, administration of XBJ injection for 5 days to 2 weeks was shown to increase overall therapeutic efficacy (Chen et al., 2020; Li et al., 2022). However, there was no significance in the incidence of complications with or without XBJ injection. Both studies suggested that XBJ injection could reduce CRP levels as well as erythrocyte sedimentation rate (ESR), TNF-α, and IL-6 levels. In severe COVID-19 patients, 7–14 days of XBJ injection could attenuate major clinical symptoms and reduce the length of ICU stay (Luo et al., 2021; Wen et al., 2020). However, it did not significantly reduce 28-day mortality or time to negative nucleic acid test. In addition to the decrease in CRP and ESR levels, these severe patients showed an increase in lymphocyte levels after XBJ injection.

Two cohort studies showed a higher 28-day discharge rate (66.7% vs. 22.2%), 28-day survival rate (91.7% vs. 81.9%), and overall efficacy (68.2% vs. 50.0%) after XBJ injection (Liu et al., 2021; Zhang et al., 2020). However, the potential mechanisms of XBJ injection have not been well discussed. A retrospective study showed that 16 days of XBJ injection shortened the time of SARS-CoV-2 RNA clearance and improved CT imaging results without causing adverse events related to liver and kidney function (Wang et al., 2021). Patients also showed reductions in CRP and serum ferritin levels. A case-control study showed that XBJ injection significantly lowered body temperature, while it was not significant in making nucleic acid tests negative (Guo et al., 2020). An observational study by Ma et al. further investigated the potential mechanisms of XBJ injection in COVID-19 treatment in 11 patients (Ma et al., 2020). They found that XBJ injection could protect cells from virus-induced cell death and reduce pro-inflammatory cytokine levels, although it was not significant for white blood cell (WBC), neutrophil count, CRP, and procalcitonin (PCT) levels. Research included 455 participants with COVID-19 showed that neutrophil to lymphocyte platelet ratio (NLPR) is the most reliable inflammatory marker for predicting prognosis among individuals with COVID-19, and can accurately identify individuals who may benefit from XBJ injection (Liao et al., 2025). A summary of the clinical trials is provided in Table 4.

**TABLE 4:** Clinical trials in XBJ injection for COVID-19 treatment.

Research type	Patients	Intervention/Control	Intervention	Control	XBJ dosage	Outcomes	Potential mechanisms	Ref.
RCT	severe COVID-19 patients	29/28	+ XBJ	Regular treatments	50 mL, q12h,14 days	1. Attenuating main clinical symptoms 2. Decrease in length of ICU hospitalization stay 3. Not significantly reducing 28-day mortality	1. Decrease in IL-6, IL-8, and TNF-α levels 2. Increase in lymphocyte levels 3. Decrease in CRP levels	Luo et al. (2021)
Retrospective study	COVID-19 patients	23/32	+ XBJ and other traditional Chinese medicine	Regular treatments	100 mL, bid, 16 days	1. Shorter time of SARS-CoV-2 RNA clearance (12 days vs. 15.5 days) 2. Improvements in CT imaging results 3. Not significant in adverse events of liver and renal functions	decrease in CRP and serum ferritin levels	Wang et al. (2021)
Case-control study	COVID-19 patients	16/16	+ XBJ	Regular treatments	100 mL, bid, ≥7 days	1. Improvements in body temperature 2. Improvements in CT imaging results 3. Not significant in nucleic acid test turning to negative	1. Decrease in IL-6 levels 2. Decrease in TNF-α and IL-10	Guo et al. (2020)
Observation study	severe COVID-19 patients	11	Regular treatments + XBJ	-	100 mL, q12h, 7 days	1. Improvements in PSI grade and PSI score 2. Improvements in lung injury	1. Protecting cells from virus-induced cell death 2. Improvements in the oxygenation index, PaCO2, and lymphocyte count 3. Decrease in TNF-α, IP-10, MIP-1β, and RANTES levels 4. Not significant in WBC, neutrophil count, CRP, and PCT	Ma et al. (2020)
RCT	severe COVID-19 patients	20/20	+ XBJ	Regular treatments	50 mL, bid, 7 days	1. Decrease in APACHE II score 2. Improvements in conditions of patients 3. Not significant in nucleic acid test turning to negative	1. Increase in white blood cell and lymphocyte counts 2. Decrease in CRP and ESR levels	Wen et al. (2020)
Cohort study	severe COVID-19 patients	72/72	+ XBJ	Regular treatments	100 mL, bid, ≥1 day	1. Improvements in PSI risk score 2. Higher 28-day discharge rate (66.7% vs. 22.2%) 3. Higher 28-day survival rate (91.7% vs. 81.9%)	not mentioned	Liu et al. (2021)
RCT	COVID-19 patients	15/15	+ XBJ	Regular treatments	100 mL, bid, 2 weeks	1. Higher overall efficiency (73.33% vs. 53.33%) 2. Not significant in occurrence of complications	decrease in CRP levels	Chen et al. (2020)
Cohort study	common COVID-19 patients	22/22	+ XBJ	Regular treatments	50 mL, bid, 7 days	1. Improvements in CT imaging results 2. Higher overall efficiency (68.2% vs. 50.0%) 3. Not significant in occurrence of complications	not mentioned	Zhang et al. (2020)
RCT	patients suspected of COVID-19	24/24	+ XBJ	Regular treatments	50 mL, bid, 5 days	1. Higher overall efficiency (95. 8% vs. 83. 3%)	1. Decrease in CRP levels 2. Decrease in ESR, TNF-α, and IL-6 levels	Li et al. (2022)
retrospective study	common COVID-19 patients	455	+ XBJ	Regular treatments	-	1. Providing XBJ injection to patients with NLPR >3.29 was associated with a lower risk of 60-day all-cause mortality	reduce in NLPR	Liao et al. (2025)

Regular treatments include nutritional support, oxygen therapy, nebulization, antibiotics, non-invasive and invasive ventilation if necessary, resolving phlegm and cough, and maintaining electrolyte balance. NLPR, neutrophil to lymphocyte platelet ratio.

Based on these clinical trials, a meta-analysis was performed to evaluate the effects and safety of XBJ injection in COVID-19 patients. Sun et al. analyzed seven studies (204 patients in the XBJ group and 183 patients in the control group) and found that XBJ injection could reduce 28-day mortality, CRP, ESR, and IL-6 levels, and increase peripheral blood leukocyte and lymphocyte counts (Sun et al., 2023). Meanwhile, the side effects of XBJ injection were not obvious. However, there was no evidence that XBJ injection could improve the nucleic acid conversion rate and CT results of COVID-19 patients. Luo et al. analyzed nine studies (230 patients in the XBJ group and 227 patients in the control group) and found that XBJ injection had a higher overall effect rate and a lower 28-day mortality rate, and it could increase the lung CT recovery rate and decrease the CRP rate (Luo et al., 2022). However, there was no significant difference in nucleic acid negative conversion rate or WBC level. The ESR of patients with severe COVID-19 and other types was significantly decreased by XBJ injection. Similar to Sun’s study, the side effects of XBJ injection were not obvious compared to the control group. Taken together, these studies indicated that XBJ injection has advantages in improving therapeutic effects, reducing 28-day mortality, improving lung recovery, and reducing pro-inflammatory factors in patients with COVID-19.

## 6 Comparative analysis of XBJ injection in COVID-19 and other related conditions

XBJ has garnered significant attention for its potential therapeutic effects in treating severe infections, including COVID-19 and other related conditions, such as severe community-acquired pneumonia (SCAP) and sepsis.

XBJ’s therapeutic effects in SCAP are primarily attributed to its ability to downregulate key inflammatory pathways. For instance, XBJ has been shown to inhibit the expression of TLR4 and NF-κB, which are critical in mediating the inflammatory response (Song et al., 2022). Additionally, XBJ can modulate the balance of Th17 and T regulatory cells, thereby reducing the severity of the inflammatory cascade. These mechanisms collectively contribute to the improvement of clinical outcomes in SCAP patients.

A large multicenter randomized controlled trial showed that XBJ injection significantly improved the pneumonia severity index (PSI) risk rating and reduced the 28-day mortality rate in patients with SCAP (Song et al., 2019). Specifically, the 28-day mortality rate was reduced from 24.63% to 15.87% in patients treated with XBJ, representing an absolute reduction of 8.76%. Furthermore, XBJ administration led to a shorter duration of mechanical ventilation and ICU stay, with median values of 11.0 days and 12 days, respectively, compared to 16.5 days and 16 days in the placebo group. These findings highlight the clinical benefits of XBJ injection in managing SCAP.

In sepsis, XBJ injection exerts its therapeutic effects through multiple pathways. It protects endothelial cells, improves microcirculation, and alleviates coagulopathy, which are critical in preventing the progression of organ dysfunction (Song et al., 2022). XBJ also downregulates the expression of HMGB1 and RAGE, key mediators of sepsis-induced organ injury. Moreover, it promotes the polarization of macrophages towards the M2 phenotype, which is associated with tissue repair and anti-inflammatory effects. These mechanisms collectively contribute to the mitigation of the systemic inflammatory response syndrome (SIRS) and improve survival rates in sepsis patients.

The EXIT-SEP trial, a large-scale randomized controlled trial, showed that adjunctive therapy with XBJ injection significantly reduced 28-day mortality in sepsis patients (Lou et al., 2025). Post-hoc analysis of this trial further revealed that XBJ was particularly effective in patients with respiratory dysfunction, acidosis, and shock. These findings suggest that XBJ injection can be a valuable adjunctive therapy in specific sepsis subgroups, potentially improving treatment efficiency and reducing healthcare costs.

Therefore, XBJ injection has shown promising therapeutic effects in both SCAP and sepsis through its anti-inflammatory and immune-modulating properties. Clinical trials have showed significant improvements in key outcomes, such as mortality rates, duration of mechanical ventilation, and ICU stay. Future research should focus on further elucidating the specific mechanisms of action and identifying patient subgroups that are most likely to benefit from XBJ injection. This approach could pave the way for more personalized and effective treatment strategies in managing severe infections.

## 7 Discussion

Discrepancies in the pharmacological effects of XBJ injections for treating SARS-CoV-2 infections, as observed in clinical studies, are due to various factors. Some studies show significant improvements in certain indicators, while others do not (Ji et al., 2025). These factors include differences in study design. For example, RCTs versus cohort or retrospective studies may introduce selection bias and confounding factors. Sample size also plays a crucial role; smaller studies may lack the statistical power to detect significant effects, whereas larger studies are more likely to reveal such improvements. Additionally, patient population heterogeneity, including variations in disease severity, comorbidities, and prior treatments, can influence the observed outcomes. The duration and dosage of the XBJ injection used in the studies may also contribute to inconsistent results because different regimens could affect the therapeutic response.

Furthermore, the clinical significance of the findings should be carefully interpreted. While some studies report improvements in indicators such as inflammatory markers (e.g., C-reactive protein and interleukin-6) and clinical symptoms, the impact on long-term patient outcomes, such as mortality and quality of life, is unclear (Lin et al., 2025). Research limitations include publication bias, whereby studies with positive results are more likely to be published, as well as reliance on non-blinded assessments, which could introduce observer bias. The generalizability of the findings may also be limited by the specific patient populations and settings in which the studies were conducted. Future research should address these limitations by conducting large-scale, multicenter, rigorously designed trials to provide definitive evidence on the effects and safety of using the XBJ injection to treat SARS-CoV-2 patients.

A comprehensive evaluation of any therapeutic intervention, including the XBJ injection, requires an understanding of its associated benefits and risks (Li et al., 2025). Regarding the treatment of SARS-CoV-2 infection, existing research has primarily examined the XBJ injection’s effects and mechanisms. However, potential adverse reactions and drug interactions have not been extensively explored (Hu et al., 2025). This knowledge gap significantly impacts clinicians’ and researchers’ ability to make informed decisions about using XBJ injections.

Adverse reactions to the injection may include allergic responses, gastrointestinal disturbances, and other systemic effects (Bin et al., 2025). However, such occurrences have not been widely reported in the existing literature. Clinicians and researchers must also consider potential interactions between XBJ and antiviral medications or other treatments commonly used to manage the disease. These interactions could affect the pharmacokinetics or pharmacodynamics of XBJ or concomitant medications, leading to suboptimal therapeutic outcomes or an increased risk of adverse effects.

In the published clinical trials, no serious adverse reaction events were observed. A few patients reported mild allergic reactions and gastrointestinal discomfort, but these reactions were transient and did not significantly affect the treatment. In combination with the research on the material basis, we analyzed the components that may pose safety risks. For example, ferulic acid, as a phenolic compound, may have certain cytotoxicity at high doses (Sun et al., 2025). However, the content of ferulic acid in XBJ injection is far below the dose that may cause toxicity. In addition, we also conducted safety assessments on other components and believe that the safety of XBJ injection is relatively high at the current dosage. Although no serious adverse reactions have been found in the current clinical studies, there are still shortcomings in safety research. For example, there is a lack of long-term safety monitoring data and insufficient research on the control of impurities. We suggest that long-term safety monitoring studies should be carried out in the future, focusing on the potential risks of long-term use of XBJ injection. At the same time, the control of impurities should be strengthened to ensure the quality and safety of the product.

Despite the promising findings from various studies, several limitations and challenges remain. Many of the *in vitro* studies lack validation through *in vivo* and clinical trials. Additionally, the dose range tested, minimal active concentration, and the use of appropriate controls are often not clearly reported, which limits the reliability of the findings. It should be emphasized that network analysis is a hypothesis-generating tool rather than definitive evidence of pharmacological mechanism. All predicted targets and pathways require experimental validation before any mechanistic claims can be made.

To address these concerns, future studies should incorporate detailed monitoring and reporting of adverse events associated with XBJ injections. This would involve conducting prospective trials with robust safety endpoints and pharmacovigilance metabolites. Investigating the potential for drug interactions through *in vitro* studies and clinical pharmacokinetic evaluations would also provide valuable insights into the safe and effective use of XBJ injections in combination with other treatments.

While PubMed is a comprehensive and widely-used database for biomedical research, our reliance on a single database may introduce selection bias. PubMed’s focus on English-language articles and its specific indexing criteria could potentially exclude relevant studies published in other languages or indexed in other databases such as Embase, Web of Science, Scopus, Cochrane Library, or CNKI. Future research should consider a broader range of databases to ensure a more comprehensive evaluation of the clinical efficacy and safety of XBJ injection in the treatment of COVID-19.

## 8 Conclusion and perspectives

XBJ injection, derived from traditional Chinese medicine, has emerged as a promising therapeutic option for COVID-19 treatment. This study provides a comprehensive synthesis of the key findings related to the material basis, mechanisms of action, clinical efficacy, and safety profile of XBJ injection.

The related metabolites of XBJ injection have been extensively characterized through various analytical techniques, including HPLC and MS. Major metabolites identified include danshensu, hydroxysafflor yellow A, paeoniflorin, ferulic acid, and senkyunolide I, among others. These metabolites exhibit a range of pharmacological activities, such as anti-inflammatory, antioxidant, and antiviral properties, which contribute to the therapeutic effects of XBJ injection in treating COVID-19.

Network analysis and molecular docking were employed to predict potential targets and explore preliminary associations between XBJ injection and COVID-19-related pathways. Key molecular targets identified include TNF, MAPK1, CASP3, EGFR, IL1B, JUN, MAPK8, MPO, PTGS2, RELA, TP53, and AKT1. These targets might be involved in pathways of COVID-19 pathogenesis, such as inflammation, immune response, and cell survival. XBJ injection might modulate these pathways by inhibiting pro-inflammatory cytokines, preventing excessive immune activation, and promoting tissue repair. While network analysis suggests possible interactions with inflammatory pathways, these remain speculative and must be confirmed through rigorous pharmacological and clinical studies.

Clinical trials have showed the therapeutic efficacy of XBJ injection in COVID-19 patients. Studies show that XBJ injection, when used in combination with standard therapy, significantly improves overall efficacy, reduces 28-day mortality, and enhances lung CT recovery. Meta-analyses of multiple studies further support these findings, indicating that XBJ injection can reduce inflammatory markers such as CRP and IL-6 levels, while improving clinical outcomes.

The safety profile of XBJ injection is also noteworthy. Clinical trials and observational studies have consistently reported a lack of significant adverse effects associated with XBJ injection. No severe adverse events related to liver or kidney function were observed, suggesting that XBJ injection is well-tolerated in COVID-19 patients. This favorable safety profile, combined with its therapeutic benefits, makes XBJ injection a potentially valuable addition to the treatment regimen for COVID-19.

While the current evidence supports the therapeutic potential of XBJ injection in COVID-19 treatment, further research is needed to fully elucidate its mechanisms of action and optimize clinical application. Large-scale, multicenter, randomized controlled trials are warranted to provide more definitive evidence on the effects and safety of XBJ injection. Additionally, studies should focus on identifying patient populations most likely to benefit from XBJ injection and exploring potential drug interactions.

In summary, XBJ injection might have significant potential in improving clinical outcomes and reducing mortality in COVID-19 patients. Its multi-target mechanism of action, predicted by network analysis and clinical studies, highlights its value in managing the complex pathophysiology of COVID-19. Future research should continue to explore the therapeutic potential of XBJ injection and its role in the broader context of COVID-19 treatment strategies.
